# Evolution and Functional Analysis of *orf1* Within *nif* Gene Cluster from *Paenibacillus graminis* RSA19

**DOI:** 10.3390/ijms20051145

**Published:** 2019-03-06

**Authors:** Qin Li, Xiaomeng Liu, Haowei Zhang, Sanfeng Chen

**Affiliations:** State Key Laboratory for Agrobiotechnology, Key Laboratory of Soil Microbiology of Agriculture Ministry and College of Biological Sciences, China Agricultural University, Beijing 100193, China; lqliqin1@126.com (Q.L.); wsw_lxm2012@163.com (X.L.); zhanghw0614@126.com (H.Z.)

**Keywords:** molecular evolution, nitrogen fixation, *orf1*, oxygen

## Abstract

*Paenibacillus* is a genus of Gram-positive, facultative anaerobic and endospore-forming bacteria. Genomic sequence analysis has revealed that a compact *nif* (nitrogen fixation) gene cluster comprising 9–10 genes *nifBHDKENX*(*orf1*)*hesAnifV* is conserved in diazotrophic *Paenibacillus* species. The evolution and function of the *orf1* gene within the *nif* gene cluster of *Paenibacillus* species is unknown. In this study, a careful comparison analysis of the compositions of the *nif* gene clusters from various diazotrophs revealed that *orf1* located downstream of *nifENX* was identified in anaerobic *Clostridium ultunense*, the facultative anaerobic *Paenibacillus* species and aerobic diazotrophs (e.g., *Azotobacter vinelandii* and *Azospirillum brasilense*). The predicted amino acid sequences encoded by the *orf1* gene, part of the *nif* gene cluster *nifBHDKENXorf1hesAnifV* in *Paenibacillus graminis* RSA19, showed 60–90% identity with those of the *orf1* genes located downstream of *nifENX* from different diazotrophic *Paenibacillus* species, but shared no significant identity with those of the *orf1* genes from different taxa of diazotrophic organisms. Transcriptional analysis showed that the *orf1* gene was expressed under nitrogen fixation conditions from the promoter located upstream from *nifB*. Mutational analysis suggested that the *orf1* gene functions in nitrogen fixation in the presence of a high concentration of O_2_.

## 1. Introduction

Biological nitrogen fixation, the conversion of atmospheric N_2_ to NH_3_, plays an important role in the global nitrogen cycle and in world agriculture. The ability to fix nitrogen is widely, but sporadically distributed among Archaea and Bacteria which includes these families: Proteobacteria, Firmicutes, Cyanobacteria, Actinobacteria, Chloroflexi and Chlorobi [1]. Most biological nitrogen fixation is catalyzed by a molybdenum-dependent nitrogenase, which consists of two component proteins: Fe protein and MoFe protein. The MoFe protein component is an α_2_β_2_ heterotetramer (encoded by *nifD* and *nifK*) that contains two metalloclusters: FeMo-co, a [Mo-7Fe-9S-C-homocitrate] cluster, which serves as the active site of substrate binding and reduction and the P-cluster, a [8Fe-7S] cluster which shuttles electrons to FeMo-co. The Fe protein (encoded by *nifH*) is a γ_2_ homodimer bridged by an intersubunit (4Fe-4S) cluster that serves as the obligate electron donor to the MoFe protein [2,3].

Nitrogenase emerged in anaerobes and later diversified into facultative anaerobes and aerobes. The transition of nitrogenase from anaerobic to facultative anaerobic and aerobic organisms was accompanied by a substantial increase in the number of *nif* genes from a minimum of 7 to a maximum of 20 genes [4]. For example, a minimum of 7 *nif* genes (*nifHDKEBI1I2*) were identified in the mesophilic archaeon *Methanocaldococcus* sp. strain FS406-22, a *nif* gene cluster composed of 9–10 genes (*nifBHDKENX* (*orf1*) *hesAnifV*) was conserved in facultative *Paenibacillus* species, and 20 *nif* genes (*nifJHDKTYENXUSVWZMFLABQ*) were found in *Klebsiella oxytoca* [5]. The obligate aerobes *Azotobacter vinelandii* contains the most complex *nif* gene cluster composed of *nifHDKTYENXUSVZMFLABQ* as well as *nafY*, *iscA* and other function-unknown genes, which colocalize with the *nif* genes [4]. A careful comparison analysis of the repertoire of *nif* genes in known diazotrophic species demonstrates that a minimum set of six genes *nifHDKENB* coding for structural and biosynthetic components were present [1]. Genetic and biochemical studies on the two model diazotrophs *K. oxytoca* and *A. vinelandii* revealed that *nifH*, *nifD* and *nifK* genes encode the structural subunits, the *nifE*, *nifN*, *nifX*, *nifB*, *nifQ*, *nifV*, *nifY*, *nifS*, *nifU* and *nifH* contribute to the synthesis and insertion of FeMo-co into nitrogenase, and *nifL* and *nifA* are involved in the regulation of other *nif* gene transcription [3]. In archaea and some Gram-positive anaerobes, NifI1 and NifI2, the GlnB homologues, regulate nitrogenase activities at a post-transcriptional level by interacting with *nifHDK* in response to N availability [6].

*Paenibacillus* is a large genus of Gram-positive, facultative anaerobic, endospore-forming bacteria. The facultative anaerobic *Paenibacillus* is in the middle stage of evolutionary history from anaerobes to aerobes. The genus *Paenibacillus* currently comprises more than 150 named species, more than 20 of which have a nitrogen fixation ability [7,8]. Our comparative genomic analysis showed that diazotrophic *Paenibacillus* species fell into two distinct sub-groups (Sub-group I and Sub-group II). The N_2_-fixing strains (e.g., *Paenibacillus polymyxa* WLY78) within Sub-group I had a *nif* gene cluster consisting of nine genes (*nifB*, *nifH*, *nifD*, *nifK*, *nifE*, *nifN*, *nifX*, *hesA* and *nifV*) within a 10 kb region, while the N_2_-fixing strains (e.g., *Paenibacillus graminis* RSA19) within Sub-group II have a *nif* gene cluster composed of 10 genes (*nifB*, *nifH*, *nifD*, *nifK*, *nifE*, *nifN*, *nifX*, *orf1*, *hesA* and *nifV*) within a 11 kb region. The *orf1* located between *nifX* and *hesA* is the major pronounced difference in the *nif* cluster between Sub-group I and Sub-group II [8]. Each of the two *nif* gene clusters is organized as a single operon and transcribed under the σ^70^ promoter located in front of *nifB* [9,10]. Recently, our studies have revealed that GlnR simultaneously acts as an activator and a repressor for *nif* gene transcription by binding to two different loci of the single *nif* promoter region according to nitrogen availability [11]. The *orf1* located downstream of *nifENX* was also found in some other diazotrophic bacteria, such as *Azospirillum brasilense* Sp7 and *Rhodobacter capsulatus* [12,13]. Although *orf1* gene behind *nifENX* was often conserved in a lot of *nif* gene clusters of diazotrophs, its evolution and function were not fully clarified.

In this study, we aimed to investigate the evolution of *orf1* located downstream of *nifENX* during the evolutionary history of nitrogenase from anaerobes to aerobes, and determine the function of *orf1* in nitrogen fixation. We selected *P. graminis* RSA19, a gram-positive, facultative anaerobic and endospore-forming bacterium isolated from the rhizosphere of maize, for this study. Our results revealed that *orf1* located downstream of *nifENX* was originally found in the *nif* gene cluster of *Clostridium ultunense* and some *Paenibacillus* species/strains of Firmicutes and then perhaps it was transferred to other aerobes. The expression and transcription of the *orf1* gene were determined by qRT-PCR and RT-PCR. Mutation analysis showed a much lower nitrogenase activity in the Δ*orf1* mutant than in the wild-type strain, which was observed under high oxygen. These results indicated that *orf1* plays a role in the protection of the nitrogenase against inactivation by O_2_.

## 2. Results and Discussion

### 2.1. Acquisition and Inheritance of Paenibacillus orf1 Gene

The *orf1* gene is a part of the *nif* gene cluster (*nifB*, *nifH*, *nifD*, *nifK*, *nifE*, *nifN*, *nifX*, *orf1*, *hesA* and *nifV*) in *P. graminis* RSA19 and some other *Paenibacillus* species or strains. To investigate the evolution of *orf1*, we here performed a comparison of the *nif* gene clusters from the representatives of diazotrophic methanogenic archae and bacteria in six taxonomic phyla: Actinobacteria, Chlorobi, Chloroflexi, Cyanobacteria, Firmicutes and Proteobacteria.

**Figure 1:**
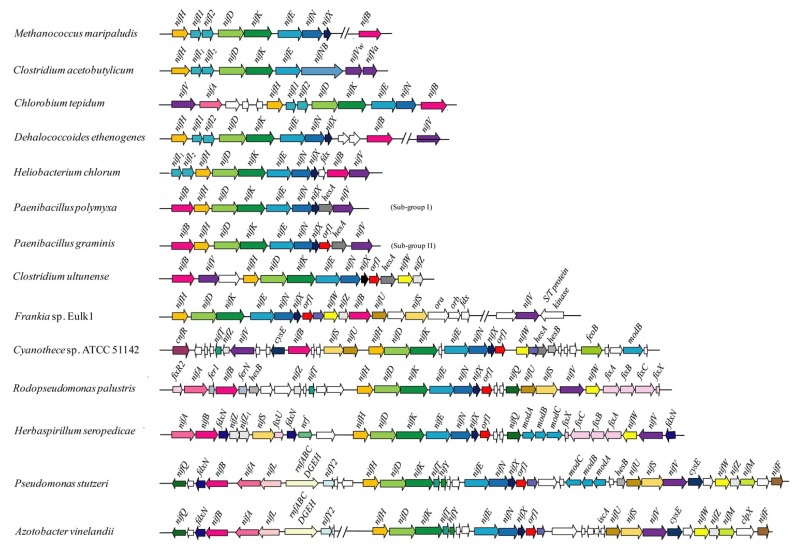
Comparison of the *nif* gene clusters from the representatives of anaerobic, facultative anaerobic, and aerobic N_2_-fixing microorganisms. Sequence of the identified regions was retrieved from GenBank and used to draw the diagram. Genes of the same color in different organisms are homologous. The *orf1* and its homologous genes are colored in red.

As shown in Figure 1, the simplest *nif* gene organization is found in methanogenic archae. For examples, *Methanocaldococcus* sp. strain FS406-22, *Methanobacterium thermoauttatrophicum*, *Methanococcus maripaludis* and *Methanosarcina mazei* Gö1, carry a single *nif* gene cluster composed of six to eight *nif* genes (*nifH*, *nifI1*, *nifI2*, *nifD*, *nifK*, *nifE*, *nifN* and *nifX*) in a single operon [4,14,15,16]. The *nifB* gene, which is essentially required for nitrogenase, is located elsewhere on genomes outside of the *nif* gene cluster in these archae. The *nifB nifH nifD nifK nifE nifN* are responsible for the synthesis of nitrogenase, and the *nifI1 nifI2* are involved in the regulation of nitrogenase activity in these diazotrophic archae [17,18].

The regulatory genes *nifI1* and *nifI2*, which were common in methanogenic archae were also found in most of the anaerobic, Gram-positive bacteria. For example, in the strictly anaerobic, Gram-positive phototrophic bacterium *Heliobacterium chlorum*, the positions of *nifI1* and *nifI2* were in the front of *nifH* in the *nif* gene cluster composed of 11 genes in the order *nifI1*, *nifI2*, *nifH*, *nifD*, *nifK*, *nifE*, *nifN*, *nifX*, *fdx*, *nifB* and *nifV* [19,20]. Notably, *nifB* emerged as a part of the *nif* gene cluster in these anaerobic bacteria. A *nif* gene cluster composed of *nifV nifA* (*orf1 orf2 orf3*) *nifH nifI1 nifI2 nifD nifK nifE nifN nifB* was found in *Chlorobium tepidum,* which is a green sulphur bacterium [21].

Both *Paenibacillus* and *Clostridium* belong to Firmicutes. *Clostridium* is a genus of obligate anaerobic bacteria, while *Paenibacillus* is a genus of facultative anaerobic bacteria. The gene organization *nifH-nifI1-nifI2-nifD-nifK-nifE-nifN*, which was common in methanogenic archae was found in anaerobic *Clostridium acetobutylicum* and *Clostridium beijerinckii* [22]. For example, a *nif* gene cluster composed of nine genes (*nifH nifI1 nifI2 nifD nifK nifE nifN-B nifV_w_ nifV_α_*) was found in *C. acetobutylicum* ATCC 824 [22].

A *nif* gene cluster consisting of nine genes within a 10 kb region in the order *nifB*, *nifH*, *nifD*, *nifK*, *nifE*, *nifN*, *nifX*, *hesA* and *nifV* was identified in diazotrophic *Paenibacillus* species. Importantly, an *orf1* emerged downstream of *nifENX* within the *nif* gene cluster in some *Paenibacillus* species [8]. The regulation genes *nifI1* and *nifI2*, which were conserved in anaerobic archae and bacteria were lost in the *nif* gene clusters of the facultative anaerobic *Paenibacillus*. In place of *nifI1* and *nifI2*, a *glnR* which is not associated with the *nif* gene cluster regulated the transcription of the *nif* gene cluster in *Paenibacillus* [11]. Interestingly, from the genome sequence data of *Clostridium ultunense* strain Esp [23], we found that a *nif* gene cluster (*nifB nifV orf1 nifH nifD nifK nifE nifN nifX orf1 hesA nifW nifZ*), which was different from those of *C. acetobutylicum* and *C. beijerinckii*, emerged in this bacterium. The organization of *nifH-nifD-nifK-nifE-nifN-nifX-orf1-hesA*, which is conserved in diazotrophic *Paenibacillus*, was for the first time found in *Clostridium*. From the hypothesis that the transition of Nif from anoxic to oxic environments, the organization of *nifH-nifD-nifK-nifE-nifN-nifX-orf1-hesA* including *orf1* in *Paenibacillus* should be evolved via horizontal gene transfer from *C. ultunense* strain Esp. However, compared to the 9 to 10 genes within the *nif* genes cluster of *Paenibacillus*, the numbers of the 13 genes within the *nif* genes cluster of *C. ultunense* were larger, and the 2 genes *nifW* and *nifZ*, which were found in aerobic diazotrophic bacteria, emerged in this bacterium. From the hypothesis that the evolution of Nif during the transition from anaerobic to aerobic metabolism was accompanied by a substantial increase in the number of *nif* genes, the organization of *nifH-nifD-nifK-nifE-nifN-nifX-orf1-hesA* including *orf1* in *C. ultunense* strain Esp should be evolved via horizontal gene transfer from *Paenibacillus*.

As shown in Figure 1, the organization *nifH-nifD-nifK-nifE-nifN-nifX-orf1*, which is conserved in facultative anaerobic *Paenibacillus*, was also identified in *Frankia* (e.g., *Frankia* sp. EulK1 [24]). The *nifHDKENXorf1* cluster was also identified, but was separated into two clusters: *nifHDK* and *ENXorf1* in Cyanobacteria (e.g., *Cyanothece* sp. 51,142 [25]) and Proteobacteria: *Rhodobacter capsulatus*, *Herbaspirillum seropedicae*, *Rhodopseudomonas palustris*, *Pseudomonas stutzeri* and *Azotobacter vinelandii*, with NifA as a positive transcription regulator in these diazotrophs [26,27,28,29].

### 2.2. Identity and Phylogenetic Analysis of the Orf1

**Figure 2:**
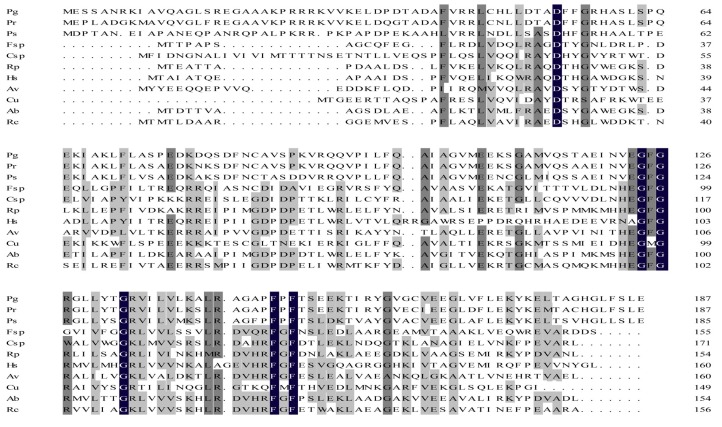
Comparison of the predicted amino acid sequences of the *Paenibacillus graminis* (Pg) Orf1 proteins with analogous gene products from *Paenibacillus sabinae* (Ps), *Paenibacillus riograndensis* (Pr), *Clostridium ultunense* (Cu), *Frankia* sp. EuIK1(Fsp), *Herbaspirillum seropedicae* (Hs), *Cyanothece* sp. ATCC 51,142 (Csp), *Rhodopseudomonas palustris* (Rp), *Rhodobacter capsulatus* (Rc), *Azotobacter vinelandii* (Av) and *Azospirillum brasilense* (Ab). A black background indicates conserved residues in all aligned sequences, dark grey indicates conserved residues in at least 75% of the aligned sequences, and light grey indicates conserved residues in at least 50% of the aligned sequences.

The *orf1* gene of *P. graminis* RSA19 is 567 bp in length. The predicted protein of Orf1 has a molecular weight of 20.5 kDa and pI of 8.43. The deduced-amino acid sequence of the *orf1* gene product in *P. graminis* RSA19 was aligned with the corresponding sequences from various known diazotrophs extracted from GenBank databases (Figure 2). The identities of Orf1 of *P. graminis* RSA19 with those from other *Paenibacillus* species at amino acid levels ranged between 60% and 90%. Except that *P. graminis* Orf1 showed the highest identities with those of other diazotrophic *Paenibacillus* spp., which showed the highest identity (32.89%) with that of *C. ultunense*. The *P. graminis* Orf1 showed 18–32% identities with those of *Frankia* sp. EuIK1, *Herbaspirillum seropedicae*, *Cyanothece* sp. ATCC 51142, *Rhodopseudomonas palustris*, *Rhodobacter capsulatus* and *Azotobacter vinelandii* (Table S1).

Structure analysis revealed that the predicated protein encoded by the *orf1* located downstream of *nifENX* in cyanobacterium *Cyanothece* 51,142 had a conserved domain (DUF269) [30]. We found that the Orf1 of *P. graminis* RSA19 and those from other diazotrophs also had the conserved domain (DUF269). Amino acid residues within this domain were conserved, such as G124, G126, G131, F146, and F148 (Figure 2). The predicted 3-dimensional structure of Orf1 protein using SWISS-MODEL online software (https://swissmodel.expasy.org.) was identical to that of Cce_0566 from *Cyanothece* (Figure S1). The DUF269 domain, which is only found in the nitrogen-fixing species may play some role in nitrogen fixation.

**Figure 3:**
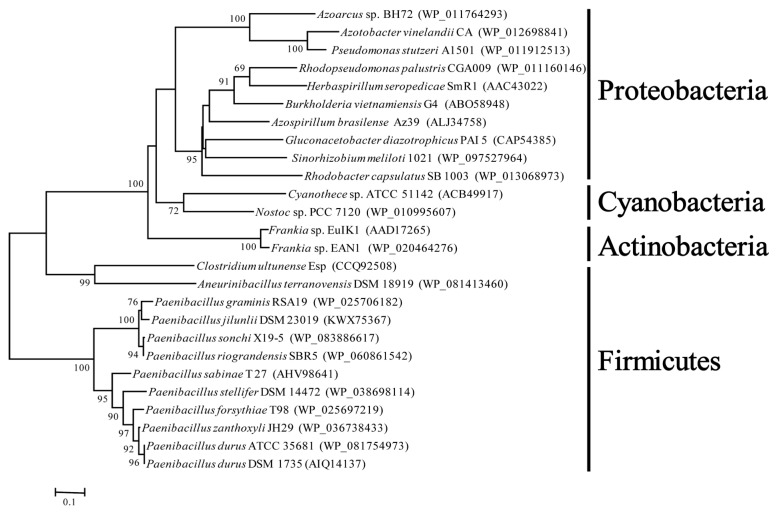
Neighbor-joining phylogenetic tree based on amino acid sequences of Orf1. Bootstrap analysis was performed with 1000 cycles. Only bootstrap values greater than 50% are shown at the branch points. Bar 0.1 substitutions per amino acid positions.

The phylogenetic analysis showed that *orf1* genes downstream of *nifENX* from facultative anaerobic *P. graminis* RSA19 and other *Paenibacillus* species (*P. sonchi*, *P. riograndensis*, *P. sabine* and *P. durus*) formed a monophyletic group, and *orf1* genes downstream of *nifENX* from aerobic bacteria, such as Frankia, Cyanobacteria and Proteobacteria, formed another group (Figure 3). The data were consistent with our previous results that *Paenibacillus* and *Frankia* were sister groups by the *NifBHDKENX* phylogenetic analysis [8]. The results suggested that the *orf1* located downstream of *nifENX* was originally found in *Paenibacillus* species and *C. ultunense,* and then perhaps was transferred to anaerobic and aerobic bacteria, such as Cyanobacteria and Proteobacteria.

### 2.3. Transcriptional Analysis of the P. graminis orf1

**Figure 4:**
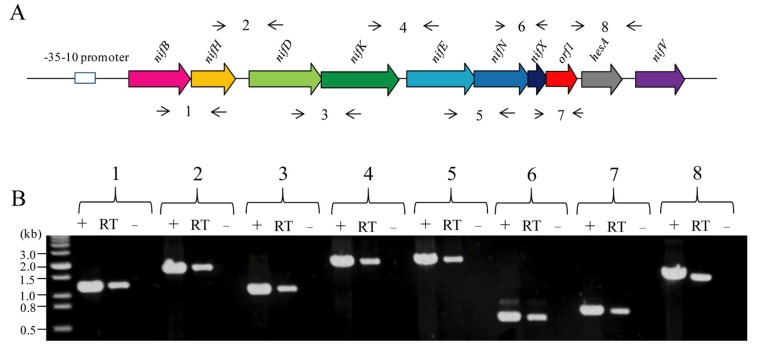
The *nif* genes of *P. graminis* RSA19 are organized in an operon as determined by RT-PCR. (**A**) Outline of the strategy. Primers used and amplified products (numbered) are given below the schematic representation of the genes. (**B**) Result of RT-PCR reactions with RNA from *P. graminis* RSA19 grown under N_2_-fixing conditions. The numbering on the top of the gels corresponds to the product numbers drawn schematically in the outline given above. RT, standard RT-PCR reaction; (−), negative control in which no reverse transcriptase was added to the RT reaction; (+), positive control in which genomic DNA was used as template in the RT-PCR.

**Figure 5:**
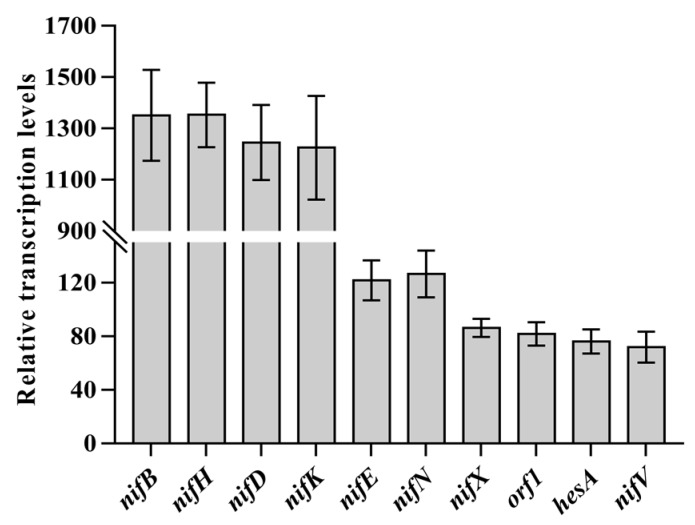
qRT-PCR analysis of transcripts of *nifBHDKENXorf1hesAnifV* genes of *P. graminis* RSA19 under N_2_-fixing conditions. All values indicate relative fold increase of transcription standardized against 16S rDNA transcript levels.

In this study, sequence analysis and RT-PCR demonstrated that the 10 genes *nifB nifH nifD nifK nifE nifN nifX orf1 hesA nifV* in *P. graminis* RSA19 were organized as an operon under the control of a -35/-10 type σ^70^-dependent promoter located upstream of *nifB* (Figure 4). Similar results that the 10 genes *nifB nifH nifD nifK nifE nifN nifX orf1 hesA nifV* in *P. sabinae* T27 are organized as an operon were reported in [10]. In this study, qRT-PCR results showed that these 10 genes within the *nif* gene operon under N_2_-fixing condition (without NH_4_^+^ and O_2_) exhibited a significant transcript ranging from 60- to 1300-fold compared to non-N_2_-fixing condition (100 mM NH_4_^+^ and 21% O_2_) (Figure 5), suggesting that *nif* gene expression in *P. graminis* RSA19 was strongly regulated by ammonium and oxygen. We found that the transcripts of *nifBHDK* gene were much higher than those of *nifENXorf1hesAnifV*, although these genes were co-transcribed from a common promoter in front of *nifB*. The different abundances of these co-transcribed genes suggested that these transcripts have different processing and stabilities, similar results were also found in *P. polymyxa* WLY78 [31] and *A. vinelandii* [32]. Taken together, the results suggest that the *orf1* gene is expressed under nitrogen fixation conditions from the promoter located upstream from *nifB* in *P. graminis* RSA19. Likewise, the *orf1* expression occurred only under nitrogen-fixing conditions in *Frankia* and *Cyanothece* 51,142 [33,34,35].

### 2.4. Mutation and Functional Analysis of the orf1 Gene

**Figure 6:**
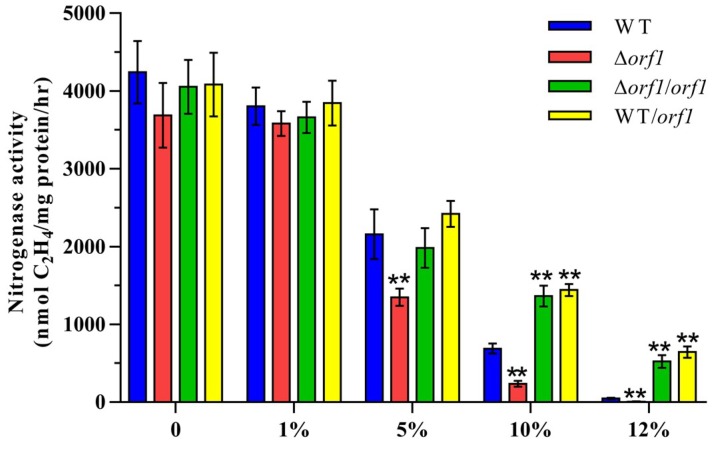
Effect of the different concentrations of O_2_ on Nitrogenase activity of *P. graminis* RSA19 (WT), Δ*orf1* (deletion mutant), Δ*orf1*/*orf1* (complementation strain) and WT/*orf1* (overexpression strain). The nitrogenase activities of these strains were assayed by the C_2_H_4_ reduction method and expressed as nmol C_2_H_4_/mg protein/hr. The nitrogenase activity of the WT was used as a control. ** indicates significant differences between the control and other strains determined by LSD at P < 0.01.

To elucidate the function of *orf1* in nitrogen fixation of *P. graminis* RSA19, we constructed an in-frame deletion mutant Δ*orf1*, a complementary strain (Δ*orf1/orf1*) and an overexpression strain (WT/*orf1*) as described in the materials and methods section. In comparison with wild-type *P. graminis* RSA19, which exhibits the highest nitrogenase activity under anaerobic and microaerobic conditions and no activity in the presence of more than 12% O_2_, activities in Δ*orf1* mutant were a little lower compared to the wild-type strain under low (0–1%) oxygen conditions (Figure 6). Especially at high concentrations of oxygen, a much lower nitrogenase activity in the Δ*orf1* mutant than in the wild-type strain was observed. Complementation and overexpression of *orf1* by introduction of *orf1* carried on multicopy vector pHY300PLK into Δ*orf1* mutant and wild-type strain led to the enhancement of activity in the presence of high oxygen. These results suggest that *orf1* is beneficial for nitrogen fixation at high oxygen concentrations. Furthermore, we found that Δ*orf1* mutant grew equally well both in the absence and presence of high oxygen concentrations. These results for the first time indicate physiological involvement of *orf1* gene products in the protection of the biosynthetic pathway or protection of the nitrogenase under high oxygen conditions.

**Figure 7:**
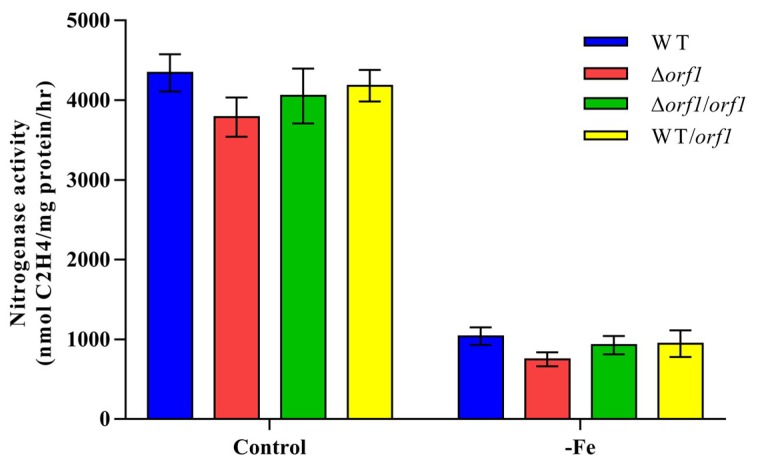
Nitrogenase activity of *P. graminis* RSA19 (WT), Δ*orf1* (deletion mutant), Δ*orf1*/*orf1* (complementation strain) and WT/*orf1* (overexpression strain) under iron limitation. These strains were grown anaerobically in a normal nitrogen-limited medium (Control) and nitrogen-limited medium without ferric citrate (-Fe). The nitrogenase activities of these strains were assayed by C_2_H_4_ reduction method and expressed as nmol C_2_H_4_/mg protein/hr.

It was previously reported that insertional mutation of the *orf1* gene of *H. seropedicae* had no effect on nitrogenase activity under a normal nitrogen-limited condition [36], but resulted in a significant decrease in nitrogenase activity under low iron levels [37]. In this study, we found that both the Δ*orf1* mutant and wild-type strain exhibited a significant reduction in nitrogenase activity under iron limitation compared to an iron sufficiency condition (Figure 7). Our results showed that *orf1* gene was not closely related to nitrogenase activity mediated by iron.

A recurrent characteristic of the location of *orf1* in many diazotrophs is the presence in their neighborhood, usually in the same operon, of *nifENX* genes involved in the iron-molybdenum cofactor biosynthesis, suggesting its possible participation in FeMoco biosynthesis. Functional analysis of *orf1* gene located downstream of *nifENX* was also performed in several diazotrophs, but obvious functions of *orf1* gene in nitrogen fixation are still lacking. For example, mutation of a ferredoxin-like gene of *R. capsulatus*, which is co-transcribed with the *nifENX* genes did not abolish its nitrogenase activity [12], but this ferredoxin-like protein may be used as an alternative electron donor or source of iron-sulfur centers. The fact that deletion of *orf1* resulted in no loss of activity under normal nitrogen fixation conditions (anaerobic or low oxygen and without ammonium) was also observed in *A. vinelandii*, *H. seropedicae* and *R. capsulatus* [12,36,38], which was in agreement with our results. Our results suggested that *orf1* gene in *P. graminis* RSA19 was required for nitrogen fixation under high oxygen conditions. How the *orf1* affects nitrogenase activity in the presence of oxygen needs to be determined in the future. Since nitrogenase is sensitive to oxygen, a variety of strategies are used to provide protection from oxygen, including consumption of excess oxygen by respiration and regulation of *nif* gene expression [39].

## 3. Materials and Methods

### 3.1. Strains, Plasmids and Growth Conditions

Bacterial strains and plasmids used in this study are summarized in Table 1. *P. graminis* [40] and derivatives were routinely grown in an LD medium (per liter contained: 2.5 g NaCl, 5 g yeast and 10 g tryptone) at 30 °C with shaking. For assays of nitrogenase activity and *nif* expression, *P. graminis* RSA19 strains were grown in nitrogen-limited media under anaerobic conditions. For measuring the effect of oxygen on nitrogenase activity, *P. graminis* and derative strains were cultivated at different concentrations of O_2_ in nitrogen-limited media. Nitrogen-limited medium contained (per liter): 10.4 g Na_2_HPO_4_, 3.4 g KH_2_PO_4_, 26 mg CaCl_2_•2H_2_O, 30 mg MgSO_4_, 0.3 mg MnSO_4_, 36 mg Ferric citrate, 7.6 mg Na_2_MoO_4_•2H_2_O, 10 mg p-aminobenzoic acid, 5 µg biotin, 2 mM glutamate and 4 g glucose as a carbon source. Iron-free medium was prepared by omitting ferric citrate from the nitrogen-limited medium.

*Escherichia coli* strains JM109 was used for regular plasmid extraction, and the Dam− Dcm−strain Trans110 was used to produce unmethylated plasmid DNA. Thermo-sensitive vector pRN5101 [41] was used for gene disruption in *P. graminis* RSA19. Shuttle vector pHY300PLK was used for complementation and overexpression experiment. When appropriate, antibiotics were added in the following concentrations: 100 μg/mL ampicillin, 12.5 μg/mL tetracycline and 5 μg/mL erythromycin for maintenance of plasmids.

### 3.2. Molecular Techniques

Plasmid and genomic DNA extraction, gel electrophoresis, restriction mapping, transformation and molecular cloning, amplification by PCR were performed by standard methods [42] or as recommended by the instructions on the products.

### 3.3. Construction of orf1 Deletion Mutant

Deletion of *orf1* was performed by a homologous recombination method. Amplification of a 931-bp DNA fragment located upstream of *orf1* using the primer set orf1UF and orf1UR and a 912-bp DNA fragment located downstream of *orf1* using the set orf1DF and orf1DR was performed (Table 2). The two fragments flanking coding region of Δ*orf1* were then fused with *Hind* III/*Bam*H Ι digested pRN5101 vector using Gibson assembly master mix (New England Biolabs, Ipswich, USA), generating a recombinant plasmid pRDorf1. The recombinant plasmid pRDorf1 was electroporated into *P. graminis* RSA19. Then, the single-crossover transformants were selected for erythromycin resistance (Em^r^). Subsequently, the double-crossover transformants were selected from the initial erythromycin transformants after several rounds of nonselective growth at 39 °C and confirmed by PCR amplification. Correct recombination was checked using primers orf1UF and orf1DR (Table 2), followed by nucleotide sequencing of the amplicon obtained.

Complementation and overexpression of *orf1* by the introduction of *orf1* under *nifB* promoter of *nif* cluster operon carried on multicopy vector pHY300PLK into *orf1* deletion mutant and wild-type strain. A 1147 bp DNA fragment containing the coding region of *orf1* and a 576 bp promoter region of *nif* operon were PCR amplified, and then the two fragments were fused together using Gibson assembly master mix. These fragments were digested with *Bam*H I/*Hind* III, and ligated into vector pHY300PLK, generating vector pHYorf1. The recombinant plasmid was transformed into Δ*orf1* mutant and *P. graminis* RSA19, and tetracycline-resistant (Tc^r^) transformants were selected and confirmed by PCR and sequencing. The primers used here are listed in Table 2. Plasmids were transformed to *P. graminis* RSA19 by electroporation with the methods described by Zhang et al. in [43] and Bach et al. in [44] with modifications.

### 3.4. RT-PCR and qRT-PCR Analysis

For RT-PCR, *P. graminis* RSA19 was grown in N_2_-fixing conditions (without NH_4_Cl and O_2_). For qRT-PCR, *P. graminis* RSA19 and Δ*orf1* mutant strain were grown in N_2_-fixing conditions (without NH_4_Cl and O_2_) and non-N_2_-fixing conditions (100 mM ammonium and 21% O_2_). The integrity and size distribution of the RNA was verified by agarose gel electrophoresis, and the concentration was determined spectrophotometrically. Synthesis of cDNA was carried out using RT Prime Mix according to the manufacturer’s specifications (Takara Bio, Tokyo, Japan). The *nif* gene transcripts were detected by using an RT-PCR Kit with 16S rDNA as a control. Primers for *nif* genes and 16S rDNA used for PCR are listed in Table 2. qRT-PCR was performed on Applied Biosystems 7500 Real-Time System and detected by the SYBR Green detection system with the following program: 95 °C for 15 min, 1 cycle; 95 °C for 10 s and 65 °C for 30 s, 40 cycles. The relative expression level was calculated using the 2^−ΔΔ*C*T^ method [45], where ΔΔC_T_ = (C_T_ gene of *nif* − C_T_ gene of 16S rRNA) N_2_-fixing condition − (C_T_ gene of *nif* − C_T_ gene of 16S rRNA) non-N_2_-fixing condition The C_T_ value is the cycle threshold at which the detected fluorescence crosses an arbitrarily placed threshold. Each experiment was performed in triplicate.

### 3.5. Acetylene Reduction Assays of Nitrogenase Activity

Acetylene reduction assays were performed as described previously to measure nitrogenase activity [9]. *P. graminis* RSA19 and its mutant strains were grown overnight in LD medium. The cultures were collected by centrifugation, washed three times with sterilized water and then resuspended in nitrogen-limited medium containing 2 mM glutamate as a nitrogen source to a final OD_600_ of 0.2–0.4. Then, 4 ml of the culture was transferred to a 25-mL test tube and the test tube was sealed with a rubber stopper. The headspace (21 mL) in the tube was then evacuated and replaced with argon gas. Then, C_2_H_2_ (10% of the headspace volume) was injected into the test tubes. For measuring the effect of oxygen on nitrogenase activity, O_2_ (0–12% of the headspace volume) was added at the same time. Cultures were incubated at 30 °C. C_2_H_4_ production was analyzed by gas chromatography. The nitrogenase activity was expressed as nmol C_2_H_4_/mg protein/hr. All treatments were in three replicates and all the experiments were repeated three or more times. The results of the nitrogenase activity were analyzed by analysis of the one-way analysis of variance (ANOVA) using SPSS software version 20 (SPSS Inc., Chicago, IL, USA). Means of different strains were compared using the least significant difference (LSD) at a 0.05 or 0.01 level of probability.

## 4. Conclusions

*P. graminis* RSA19 has an *orf1* gene within the *nif* gene cluster (*nifB nifH nifD nifK nifE nifN nifX orf1 hesA nifV*). The *orf1* located downstream of *nifENX* was also identified in some other facultative anaerobic *Paenibacillus* species, anaerobic *Clostridium ultunense* and aerobic diazotrophs (e.g., *Azotobacter vinelandii* and *Azospirillum brasilense*). The *orf1* emerged in anaerobes and facultative anaerobes and later recruited into aerobes during the evolution process. The *orf1* gene may play a role in protection of the nitrogenase against oxygen.

## Figures and Tables

**Table 1:** Bacterial strains and plasmids used in this study.

Strain or Plasmid	Genotype and/or Relevant Characteristics	Source or Reference
**Strains**		
*Paenibacillus graminis*		
RSA19	Wild-type strain	[40]
Δ*orf1*	*orf1* in-frame deletion mutant	This study
Δ*orf1/orf1*	Complementation strain of Δ*orf1* with *orf1* carried in plasmid pHYorf1	This study
WT/*orf1*	*orf1* overexpression strain which is a derivative of *P. graminis* RSA19 carrying an *orf1* gene in plasmid pHYorf1	This study
*E. coli*		
JM109	General cloning host; *recA1*, *endA1*, *gyrA96*, *thi-1*, *hsdR17*, *supE44*, *relA1*, Δ*(lac-proAB)*/F’[*traD36, proAB+*, *lacIq*, *lacZ*Δ*M15*]	Sangon Biotech Co.
Trans110	*rpsL* (Str R) *thr leu thi-1 lacY galK galT ara tonA tsx dam dcm sup*E44 Δ(lac-*proAB*)/F′[*traD36 proAB lacIq lacZΔ*M15]	Transgen Biotech Co.
**Plasmids**		
pHY300PLK	Multiple-copy *E. coli*-*Bacillus* shuttle vector, Tc^r^	TaKaRa
pRN5101	Temperature-sensitive *E. coli-Bacillus* shuttle vector, Em^r^	[41]
pRDorf1	*orf1* deletion vector based on pRN5101	This study
pHYorf1	*orf1* overexpression vector with *orf1* in pHY300PLK	This study

**Table 2:** Primers used in this study.

Primer Name	Sequence	Location/Target
**For qRT-PCR**		
16SF	TTTGTCGTCAGCCTCGTGTTCGTG	qRT-PCR for control (16S rDNA)
16SR	ATCCCCACCTTCCTCCGGTTTG	
RBF	GCATCCGCCATGTCACTATCACC *	qRT-PCR for *nifB*
RBR	CGCACACCTTCGTCGAACACC	
RHF	GACTCCACACGCCTGATTCTGAAC *	qRT-PCR for *nifH*
RHR	CCGCCGCACTCTACGTTGATG *	
RDF	CACTGCCACCGCTCCATGAAC *	qRT-PCR for *nifD*
RDR	CACGCAGGCTCTCATAAGTCTTGG *	
RKF	CAGTCATCTCAGCCGCCACTTC *	qRT-PCR for *nifK*
RKR	TCCAAGCCGTCGATCAGATTGTTC *	
REF	CGGTCATCCCAGTGAACAGC *	qRT-PCR for *nifE*
RER	CCCGCACTGTTCATCAGCTT *	
RNF	ACACCGCTGATTGCAGGAATGG *	qRT-PCR for *nifN*
RNR	TGCCGTGCGAATTGCTGATCC *	
RXF	GCGGTGAGCTGCTAGAACTGC *	qRT-PCR for *nifX*
RXR	GGCTGCCGAACGGAACCTTAAC *	
ROF	GACTTCAACTGCGCCGTATCTCC *	qRT-PCR for *orf1*
ROR	TCCGCTCTTCTCTTCCATGACTCC *	
RAF	GTTGAAGGAAGCGACGGTGATGG *	qRT-PCR for *hesA*
RAR	GACCAGAATCAGCTTGCCGACAC	
RVF	AGGAGGATATTGCGGCGATTGC	qRT-PCR for *nifV*
RVR	CCGAGACAGGAATAGACACATGGC *	
**For construction of *orf1* deletion and overexpression strains**		
orf1UF	CGGCCACGATGCGTCCGGCGTAGAGGATCCGCTGGGGACCCCCTATAAG	Upstream of *orf1*
orf1UR	TAAATTTGCAGCTTGGCTTCCTCCCCTTCCC	
orf1DF	GGGGAGGAAGCCAAGCTGCAAATTTAGGATCGG	Downstream of *orf1*
orf1DR	TCATGGCGACCACACCCGTCCTGTGGATCCGCTTGCTCAACTCCGCATTC	
nifPF	TTATAACAGGAATTCCCGGGGATCCTGCTGCTTCCTCCTCATTTG	Promoter region of *nif* operon
nifPR	ACGATTCCATTTCCCACCTCCTAAAAGTAAC	
orf1F	GAGGTGGGAAATGGAATCGTCGGCTAAC	*orf1* ORF
orf1R	ATGGAAAAACGCTTTGCCCAAGCTTATCCTTCAAGGCTGAAC	

* Primers also used in the RT-PCR.

## Supplementary Materials

Supplementary Materials can be found at https://www.mdpi.com/1422-0067/20/5/1145/s1.

## Founding

This work was supported by the National Nature Science Foundation of China (Grant No. 31770083) and the Innovative Project of SKLAB (Grant No. 2017SKLAB1-1).
