# Supplementary material for: Evolution and Functional Analysis of orf1 Within nif Gene Cluster from Paenibacillus graminis RSA19
Source: Int J Mol Sci. 2019 Mar 6;20(5):1145. doi: 10.3390/ijms20051145 (PMC6429469; doi:10.3390/ijms20051145)
Supplement: Supplementary file 1 [file ijms-20-01145-s001.pdf]

Table S1. Comparison of the predicted protein encoded by the *orf1* gene from *P. graminis* RSA19 with those from other diazotrophic species at amino acid sequence level.

**Species**	**Amino acid identity (%)**
Paenibacillus sonchi	89.36% (168/188)
Paenibacillus riograndensis	88.83% (167/188)
Paenibacillus sabine	61.83% (115/186)
Paenibacillus durus	59.57% (112/188)
Frankia sp. EuIK1	26.51% (22/83)
Cyanothece sp. ATCC 51142	30% (24/80)
Rhodopseudomonas palustris	32.76% (19/58)
Azospirillum brasilense	24.06% (32/133)
Herbaspirillum seropedicae	20.47% (26/127)
Rhodobacter capsulatus	24.22% (31/128)
Azotobacter vinelandii	18.75% (24/128)
Clostridium ultunense	32.89% (49/149)

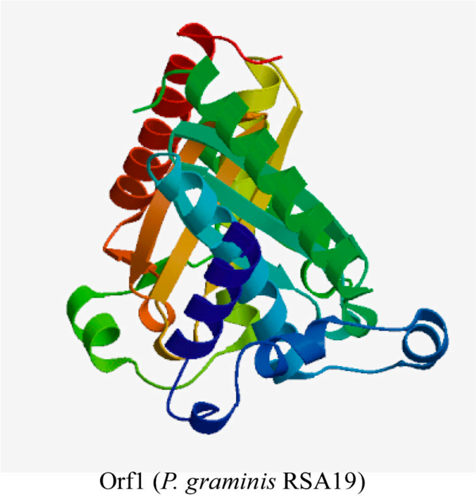

Orf1 (*P. graminis* RSA19)

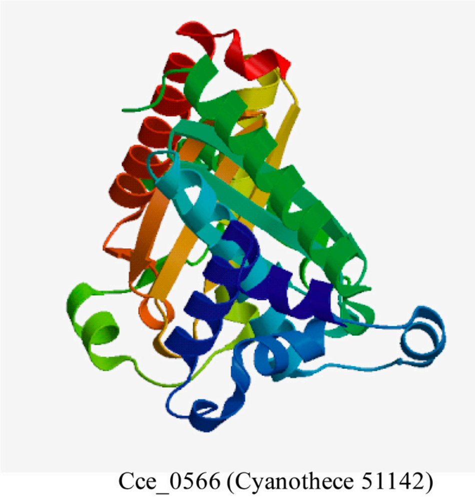

Cce_0566 (Cyanothece 51142)

Figure S1: Comparison of three-dimensional structure of *P. graminis* RSA19 Orf1 with that of Cce_0566 (DUF269) of Cyanothece 51142. The 3-dimensional structure model was predicted by SWISS-MODEL, which is an automated comparative protein modeling server.
